# Prevalence and Incidence of Nosocomial Infections in a Single Tertiary-Level Neonatal Intensive Care Unit in Oman

**DOI:** 10.7759/cureus.86936

**Published:** 2025-06-28

**Authors:** Khalid Albalushi, Abdulrahman Al Saadi, Nada Al-Rawahi, Ashfaq Khan, Amal Saki Malehi, Abdulrahman Alhadrami, Mustafa Al-Attraqchi, Widad Alessai

**Affiliations:** 1 College of Medicine and Health Science, Sultan Qaboos University, Muscat, OMN; 2 Department of Child Health, Sultan Qaboos University Hospital, Muscat, OMN; 3 Department of Pediatrics and Child Health, Royal Hospital, Muscat, OMN; 4 Department of Family Medicine and Public Health, Sultan Qaboos University, Muscat, OMN; 5 College of Medicine and Health Sciences, Sultan Qaboos University, Muscat, OMN

**Keywords:** extremely low birth weight infant, neonatal intensive care unit (nicu), nosocomial infection, oman, prevalence

## Abstract

Objectives

To determine the prevalence, incidence, and risk factors associated with nosocomial infections (NI) in neonates admitted to the Neonatal Intensive Care Unit (NICU) of Sultan Qaboos University Hospital (SQUH) in Oman.

Methods

A retrospective cohort study was conducted, involving neonates admitted to the NICU between January 2020 and December 2023. Data were collected from medical records, focusing on demographics, clinical characteristics, and laboratory results. Statistical analysis was performed to identify significant risk factors for NI. Ethical approval was obtained from the Medical Research Ethics Committee of Sultan Qaboos University.

Results

Of the 1,642 neonates admitted to the NICU at SQUH from 2020-2023, 292 developed NI. The average prevalence of NI among NICU neonates was 17.95%(N=292), with an incidence of 13.75 per 1,000 patient-days and 17.78 per 100 admissions. Respiratory tract infections are more prevalent (N=107). Gram-positive organisms are more frequent in bloodstream infections(N=47) (63.4%). However, gram-negative organisms have a higher prevalence in the urinary tract, conjunctivitis, the respiratory tract, and surgical site infections. Invasive mechanical ventilation was identified as a significant risk factor for respiratory tract infections. Additionally, respiratory tract infections were associated with prolonged hospitalizations. However, mortality rates did not differ significantly across the different infection types (P=0.49).

Conclusion

The findings underscore that NI poses a significant challenge within the NICU at SQUH, necessitating targeted interventions to mitigate its occurrence and impact on neonatal outcomes.

## Introduction

Nosocomial infections (NI) pose a formidable challenge in neonatal intensive care units globally, significantly contributing to neonatal morbidity and mortality [1,2]. NI are defined as those that develop in a patient during their stay in a healthcare setting, rather than being present or incubating at the time of admission. These infections typically manifest 48 hours or more after the patient's admission to the facility [3].

Although medical advancements have enhanced the survival and well-being of critically ill neonates, these same improvements have concomitantly increased their susceptibility to healthcare-associated infections.

The vulnerability of the neonatal immune system, combined with the common requirement for invasive medical interventions within the NICU, promotes the transmission of NI [4]. Prematurity, low birth weight, and congenital anomalies further increase the risk of neonates contracting these infections, which can lead to prolonged hospital stays, increased healthcare costs, and long-term neurodevelopmental sequelae [5]. Numerous studies worldwide have documented the concerning prevalence of NI in NICU settings, underscoring the urgent need for effective preventative measures [2,6-10]. Despite the global concern regarding NI, comprehensive data on the prevalence, incidence, and risk factors in Omani neonatal intensive care units are scarce, hindering the development of targeted prevention strategies. The available literature primarily focuses on global trends, highlighting the significant burden of these preventable infections on neonatal health outcomes [11]. Therefore, a deeper understanding of the local epidemiology is needed to develop and implement effective infection prevention and control strategies suited to the Omani healthcare context.

This study aims to determine the prevalence and incidence of NI within the NICU of SQUH, a tertiary referral center in Oman, and to identify the key risk factors contributing to their occurrence. Additionally, the study will seek to identify the most prevalent type of NI and the most common causative organism within the NICU setting.

## Materials and methods

Study subjects

This retrospective cohort study was conducted a the NICU of Sultan Qaboos University Hospital (SQUH), a tertiary care facility in Muscat, Oman. The NICU of SQUH is a 24-bed referral center for critically ill neonates from across the country, with an average of 400 annual admissions. We conducted a comprehensive retrospective review of electronic medical records, encompassing all neonates admitted to the NICU between January 2020 and December 2023, who survived beyond 48 hours of life, including those who died after this period. We excluded those who were transferred before 48 hours of life or those who died. We also excluded neonates with positive cultures obtained within the first 48 hours of admission.

Data collection

Data collection was carried out by a team of trained medical students, who gathered detailed information on demographics, clinical characteristics, and infection-related parameters. Demographic variables, such as gestational age, birth weight, gender, and mode of delivery, were extracted. Relevant clinical data, including Apgar scores, underlying medical conditions, and the use of invasive procedures, such as mechanical ventilation, central venous catheters, and parenteral nutrition, were collected to assess potential risk factors. The primary outcome of interest is the occurrence of NI, defined according to established criteria from the Centers for Disease Control and Prevention National Healthcare Safety Network [12]. Specifically, we identified infections that manifested more than 48 hours after admission to the NICU, including bloodstream infections, pneumonia, urinary tract infections, surgical site infections, conjunctivitis, and meningitis. We did not exclude any cases due to missing data.

Ethical approval was obtained from the Medical Research Ethics Committee of Sultan Qaboos University, ensuring adherence to ethical principles and patient data confidentiality throughout the study (REF. NO. SQU-EC/ 029\2024.MREC #3236).

The study did not require informed consent due to its retrospective nature.

Statistical analysis

Categorical variables described by frequency (percentage) and quantitative variables were also provided by the median (min-max). Kolmogorov-Smirnov’s normality test was performed on quantitative variables. The chi-square test was used to evaluate the categorical variables between different infection groups. The Kruskal-Wallis test was used to compare quantitative variables between different infection groups. The significant variables of univariate analysis are considered eligible covariates for univariate binary logistic regression. Univariate binary logistic regression was implemented to investigate the association between the potential risk factors and each type of nosocomial infection separately (crude OR). The Kaplan-Meier curve and the log-rank test were used to compare the survival rate among the types of NI. A P-value less than 0.25 is considered significant in the univariate analysis and will enter the multiple regression analysis (adjusted OR). The P-value less than 0.05 was considered statistically significant for other tests. All statistical analysis was performed using SPSS version 29 (IBM Corp., Armonk, NY, USA).

## Results

A total of 1,642 neonates were admitted to the NICU at SQUH between January 2020 and December 2023, of whom 292 developed NI. Respiratory tract infection was the most prevalent type of NI (N=107) among neonates admitted to the NICU. In contrast, the lowest prevalence of NI was in the central nervous system (N=2). The average overall prevalence of NI among neonates admitted to the NICU was 17.95% (14.66% in 2020, 13.53% in 2021, 24.64% in 2022, and 18.97% in 2023), with an average incidence of 13.75 NI per 1,000 patient-days and 17.78 NI per 100 admissions.

Table 1 provides a comparative analysis of baseline and demographic characteristics across the different types of NI. The results revealed significant differences in gestational age and birth weight among the infection types (P < 0.001). Neonates with respiratory tract infections had the lowest median gestational age (median=27) and N=69 (64.5%) falling within the 24-28 week range. Consequently, the median birth weight in this group was 800 grams, and N=68 (63.6%) had a birth weight of less than 999 grams. The neonates with surgical site infections (wound swabs) showed a higher prevalence of chromosomal anomalies, with nine cases observed (36.0%). The assessment of clinical variables, including invasive and non-invasive ventilation, intravenous (IV) access, total parental nutrition (TPN), and prior surgeries, revealed significant differences among the different types of nosocomial infections (P<0.05). Furthermore, the data shows a significant association between infections and cardiovascular (CVS) co-morbidities (P < 0.001). Specifically, CVS co-morbidities were more prevalent in patients with urinary tract infections (63.6%).

**Table 1:** Results of univariate analysis of potential risk factors and all types of NI NI: nosocomial infections * chi-square test

NI								Test statistic value	P-value^*^
Variables	Subgroups	Bloodstream infection (N=71)	Urinary tract infection (N=22)	Central nervous system (N=2)	Conjunctivitis (N=65)	Respiratory tract infection (N=107)	Surgical site infection (wound swabs) (N=25)		
Sex	male	44 (62.0)	13 (59.1)	1 (50)	39 (60.0)	71 (66.4)	12 (48)	3.23	0.67
	female	27 (38.0)	9 (40.9)	1 (50)	46 (40.0)	36 (33.6)	13 (52)		
Gestational age (weeks)	24-28	30 (42.3)	4 (18.2)	0 (0)	19 (29.2)	69 (64.5)	4 (16.0)	51.42	<0.001
	29-32	14 (19.7)	9 (40.9)	1 (50)	23 (35.4)	15 (14.0)	6 (24.0)		
	33-36	7 (9.9)	3 (13.6)	0 (0)	12 (18.5)	11 (10.3)	7 (28.0)		
	≥ 37	20 (28.2)	6 (27.3)	1 (50)	11 (16.9)	12 (11.2)	8 (32.0)		
Birth weight (grams)	<999	25 (35.2)	4 (18.2)	0 (0)	13 (20.0)	68 (63.6)	5 (20.0)	67.41	<0.001
	1000-1499	10 (14.1)	7 (31.8)	1 (50)	19 (29.2)	5 (4.7)	3 (12.0)		
	1500-2499	18 (25.4)	3 (13.6)	1 (50)	22 (33.8)	24 (22.4)	10 (40.0)		
	≥2500	18 (25.4)	8 (36.4)	0 (0)	11 (16.9)	10 (9.3)	7 (28.0)		
Type of delivery	Normal	35 (49.3)	11 (50.0)	2 (100)	37 (56.9)	43 (40.2)	43 (56.0)	7.53	0.18
	LSCS	36 (50.7)	11 (50.0)	0 (0)	28 (43.1)	64 (59.8)	11 (44.0)		
Chromosomal anomalies	Yes	11 (15.5)	3 (13.6)	0 (0)	11 (16.9)	8 (7.5)	9 (36.0)	14.39	0.01
	None	60 (84.5)	19 (86.4)	2(100)	54 (83.1)	99 (92.5)	16 (64.0)		
Invasive ventilation	Yes	53 (74.6)	9 (40.9)	1 (50.0)	33 (50.8)	101 (94.4)	13 (52.0)	57.28	<0.001
	None	18 (25.4)	13 (59.1)	1 (50.0)	32 (49.2)	6 (5.6)	12 (48.0)		
Non-invasive ventilation	Yes	59 (83.1)	15 (68.2)	2(100)	59 (90.8)	95 (88.8)	18 (72.0)	11.60	0.04
	None	12 (16.9)	7 (31.8)	0 (0)	6 (9.2)	12 (11.2)	7 (28.0)		
Cardiovascular co-morbidities	Yes	35 (49.3)	7 (31.8)	2(100)	17 (26.2)	68 (63.6)	10 (40.0)	27.56	<0.001
	None	36 (50.7)	15 (68.2)	2(100)	48 (73.8)	39 (36.4)	15 (60.0)		
Necrotizing Enterocolitis	Yes	18 (25.4)	3 (13.6)	0 (0)	10 (15.4)	31 (29.0)	4 (16.0)	6.94	0.23
	None	53 (74.6)	19 (86.4)	2(100)	55 (84.6)	76 (71.0)	21 (84.0)		
Hypoxic-ischaemic encephalopathy	Yes	1 (1.4)	0 (0)	0 (0)	0 (0)	4 (3.7)	1 (4.0)	3.99	0.55
	None	70 (98.6)	22 (100)	2 (100)	65 (100)	103 (96.3)	24 (96.0)		
Intraventricular hemorrhage (IVH)	Yes	25 (35.2)	5 (22.7)	1 (50.0)	22 (33.8)	48 (44.9)	8 (32.0)	75.18	0.36
	None	46 (64.8)	17 (77.3)	1 (50.0)	43 (66.2)	59 (55.1)	17 (68.0)		
Surgery	Yes	16 (22.5)	4 (18.2)	0 (0)	9 (13.8)	38 (35.5)	16 (64.0)	28.12	<0.001
	No	55 (77.5)	18 (81.8)	2(100)	56 (86.2)	69 (64.5)	9 (36.0)		
Total parental nutrition	Yes	47 (66.2)	16 (72.7)	1 (50.0)	43 (66.2)	98 (91.6)	10 (40.0)	36.81	<0.001
	No	24 (33.8)	6 (27.3)	1 (50.0)	22 (33.8)	9 (8.4)	15 (60.0)		
Intravenous accesses	Peripheral line	21 (29.6)	9 (40.9)	1 (50.0)	20 (30.8)	9 (8.4)	10 (40.0)	41.83	<0.001
	Central lines	50 (70.4)	13 (59.1)	1 (50.0)	45 (69.2)	98 (91.6)	15 (60.0)		

The distribution of causative organisms across different types of NI is presented in Table 2. Among gram-positive organisms, *Staphylococcus epidermidis *is the most prevalent causative agent in bloodstream infections (66.67%) and urinary tract infections (12.96%). Among gram-negative organisms, *Stenotrophomonas maltophilia* and *Acinetobacter baumannii *have a higher frequency than other organisms in respiratory tract infections. Also, *Klebsiella pneumonia* (40.74%) and *Pseudomonas aeruginosa* (32.08%) have a higher frequency than other organisms in conjunctivitis. The analysis revealed a significant association between the type of NI and the causative organisms (P <0.001). Gram-positive bacteria were more prevalent in bloodstream infections.

**Table 2:** Distribution of organisms according to the type of NI NI: nosocomial infections

NI							
Types of Infection		Bloodstream Infection (N=71)	Urinary tract Infection (N=22)	Central nervous system infection (N=2)	Conjunctivitis (N=65)	Respiratory tract infection (N=107)	Surgical site infection (wound swabs) (N=25)
Gram-Positive Organisms	Staphylococcus epidermidis	36 (66.67)	7 (12.96)	0 (0.0)	4 (7.41)	5 (9.26)	2 (3.70)
	Enterococcus faecalis	2 (66.67)	0 (0.0)	0 (0.0)	0 (0.0)	0 (0.0)	1 (33.33)
	Listeria monocytogenes	1 (100.0)	0 (0.0)	0 (0.0)	0 (0.0)	0 (0.0)	0 (0.0)
	Group B streptococcus	6 (75.0)	0 (0.0)	2 (25.0)	0 (0.0)	0 (0.0)	0 (0.0)
	Methicillin-Resistant Staphylococcus Aureus	0 (0.0)	0 (0.0)	0 (0.0)	3 (75.0)	1 (25.0)	0 (0.0)
	Curtobcterium albidum	1 (100.0)	0 (0.0)	0 (0.0)	0 (0.0)	0 (0.0)	0 (0.0)
	Micrococcus luteus	1 (100.0)	0 (0.0)	0 (0.0)	0 (0.0)	0 (0.0)	0 (0.0)
	Lactobacillus crispatus	0 (0.0)	0 (0.0)	0 (0.0)	0 (0.0)	1 (100.0)	0 (0.0)
	Streptococcus agalactiae	2 (100.0)	0 (0.0)	0 (0.0)	0 (0.0)	0 (0.0)	0 (0.0)
Total		47 (64.38)	7 (9.59)	2 (2.74)	7 (9.59)	7 (9.59)	3 (4.11)
Gram-Negative Organisms	Klebsiella pneumonia	7 (25.93)	0 (0.0)	0 (0.0)	11 (40.74)	7 (25.93)	2 (7.40)
	Pseudomonas aeruginosa	3 (5.66)	14 (26.42)	0 (0.0)	17 (32.08)	15 (28.30)	4 (7.54)
	Enterobacter cloacae	1 (14.29)	0 (0.0)	0 (0.0)	1 (14.29)	3 (42.85)	2 (28.57)
	Escherichia coli	8 (30.77)	0 (0.0)	0 (0.0)	5 (19.23)	9 (34.62)	4 (15.38)
	Acinetobacter baumannii	0 (0.0)	0 (0.0)	0 (0.0)	3 (17.65)	14 (82.35)	0 (0.0)
	Serratia marcescens	0 (0.0)	0 (0.0)	0 (0.0)	1 (100.0)	0 (0.0)	0 (0.0)
	Stenotrophomonas maltophilia	0 (0.0)	0 (0.0)	0 (0.0)	0 (0.0)	7 (100.0)	0 (0.0)
	Citrobacter freundii	0 (0.0)	0 (0.0)	0 (0.0)	0 (0.0)	1 (100.0)	0 (0.0)
	Elizabethkingia meningoseptica	0 (0.0)	0 (0.0)	0 (0.0)	0 (0.0)	1(100.0)	0 (0.0)
Total		21 (14.79)	14 (9.86)	0 (0.0)	38 (26.76)	57 (40.14)	12 (8.45)
Fungal	Candida	2 (22.22)	1 (11.11)	0 (0.0)	1 (11.11)	3 (33.34)	2 (22.22)
Multiple organisms		1 (1.47)	0 (0.0)	0 (0.0)	19 (27.94)	40 (58.82)	8 (11.77)

Table 3 presents the findings from the investigation of potential risk factors associated with bloodstream infection. The results indicate that there are no statistically significant variables that influence the occurrence of bloodstream infection. Similarly, the findings from the multiple binary logistic regression analyses in Table 4 reveal that there are no significant variables that impact the development of urinary tract infections.

**Table 3:** Results of the multivariate analysis of potential risk factors and bloodstream infection * Reference category

Variables	Subgroups	Crude Odds Ratio (95% CI)	P-value	Adjusted Odds Ratio (95% CI)	P-value
Gestational age (weeks)	24-28	0.59 (0.30 - 1.17)	0.13	1.55 (0.33 - 7.20)	0.58
	29-32	0.49 (0.22 – 1.01)	0.08	0.74 (0.19 – 2.96)	0.67
	33-36	0.40 (0.15 – 1.07)	0.06	0.42 (0.12 – 1.52)	0.19
	≥ 37^*^	-----	-----	-----	-----
Birth weight (grams)	<999	0.56 (0.27 - 1.14)	0.11	0.49 (0.10 - 2.36)	0.37
	1000-1499	0.57(0.23 - 1.41)	0.22	0.81 (0.17 – 3.89)	0.80
	1500-2499	0.60(0.28 - 1.30)	0.20	0.97 (0.30 - 3.15)	0.97
	≥2500^*^	-----	-----	-----	-----
Chromosomal anomalies	Yes	1.12 (0.53 – 2.37)	0.76	-----	-----
	None^*^	-----	-----	-----	-----
Invasive ventilation	Yes	1.20 (0.65 – 2.21)	0.56	-----	-----
	None^*^	-----	-----	-----	-----
Non-invasive ventilation	Yes	0.83 (0.40 – 1.72)	0.62	-----	-----
	None^*^	-----	-----	-----	-----
Cardiovascular comorbidities	Yes	1.13 (0.66 – 1.94)	0.65	-----	-----
	None^*^	-----	-----	-----	-----
Surgery	Yes	0.67 (0.36 – 1.25)	0.21	0.73 (0.38 - 1.38)	0.33
	None^*^	-----	-----	-----	-----
Intravenous accesses	Peripheral line	1.47 (0.81 – 2.69)	0.21	1.27 (0.53 – 3.08)	0.59
	Central lines^*^	-----	-----	-----	-----
Total parental nutrition	Yes	0.62 (0.35 – 1.10)	0.10	0.73 (0.32 - 1.71)	0.47
	None^*^	-----	-----	-----	-----

**Table 4:** Results of the multivariate analysis of potential risk factors and urinary tract infection * Reference category

Variables	Subgroups	Crude Odds Ratio (95% CI)	P-value	Adjusted Odds Ratio (95% CI)	P-value
Gestational age (weeks)	24-28	0.28 (0.08 – 1.05)	0.06	1.92 (0.08 – 48.89)	0.69
	29-32	1.32 (0.44 – 3.96)	0.62	8.06 (0.51 – 127.72)	0.14
	33-36	0.70 (0.17 – 2.99)	0.63	2.14 (0.34 – 13.61)	0.42
	≥ 37^*^	-----	-----	-----	-----
Birth weight (grams)	<999	0.21 (0.06 – 0.72)	0.01	0.45 (0.02 – 11.83)	0.63
	1000-1499	1.06 (0.35 – 3.19)	0.92	0.50 (0.03 – 9.30)	0.64
	1500-2499	0.23 (0.06 – 0.91)	0.04	0.11 (0.01 – 1.13)	0.06
	≥2500^*^	-----	-----	-----	-----
Invasive ventilation	Yes	0.24 (0.10 – 0.58)	0.002	0.36 (0.11 – 1.19)	0.10
	None^*^	-----	-----	-----	-----
Non-invasive ventilation	Yes	0.34 (0.13 – 0.89)	0.03	0.37 (0.12 – 1.15)	0.09
	None^*^	-----	-----	-----	-----
Cardiovascular comorbidities	Yes	0.50 (0.20 – 1.27)	0.15	0.90 (0.31 – 2.63)	0.84
	None^*^	-----	-----	-----	-----
Surgery	Yes	0.54 (0.18 – 1.64)	0.28	-----	-----
	None^*^	-----	-----	-----	-----
Intravenous accesses	Peripheral line	2.37 (0.97 – 5.81)	0.06	1.76 (0.39 – 8.04)	0.47
	Central lines^*^	-----	-----	-----	-----
Total parental nutrition	Yes	0.95 (0.36 – 2.53)	0.92	-----	-----
	None^*^	-----	-----	-----	-----

Invasive ventilation and cardiovascular co-morbidities were associated with a lower risk of conjunctivitis infection, with odds ratios of 0.39 for both variables. This suggests the risk of conjunctivitis infection was reduced as compared to other types of NI in relation to the factors shown in Table 5.

**Table 5:** Results of the multivariate analysis of potential risk factors and conjunctivitis infection * Reference category

Variables	Subgroups	Crude Odds Ratio (95% CI)	P-value	Adjusted Odds Ratio (95% CI)	P-value
Gestational age (weeks)	24-28	0.76 (0.34 – 1.72)	0.51	2.82 (0.42 – 12.33)	0.34
	29-32	2.18 (0.96 – 4.99)	0.06	1.20 (0.26 – 5.58)	0.82
	33-36	1.83 (0.71 – 4.70)	0.21	2.34 (0.62 – 8.73)	0.21
	≥ 37^*^	-----	-----	-----	-----
Birth weight (grams)	<999	0.50 (0.27 – 1.20)	0.12	0.45 (0.08 – 2.53)	0.37
	1000-1499	2.86 (1.75 – 6.94)	0.02	2.89 (0.55 – 15.11)	0.21
	1500-2499	1.54 (0.67 – 3.51)	0.31	1.41 (0.40 – 4.98)	0.59
	≥2500^*^	-----	-----	-----	-----
Chromosomal anomalies	Yes	1.29 (0.61 – 2.73)	0.51	-----	-----
	None^*^	-----	-----	-----	-----
Invasive ventilation	Yes	0.29 (0.16 – 0.52)	<0.001	0.39 (0.17 – 0.88)	0.02
	None^*^	-----	-----	-----	-----
Non-invasive ventilation	Yes	1.98 (0.80 – 4.91)	0.14	2.62 (0.94 – 7.29)	0.07
	None^*^	-----	-----	-----	-----
Cardiovascular comorbidities	Yes	0.32 (0.17 – 0.58)	<0.001	0.39 (0.19 – 0.77)	0.007
	None^*^	-----	-----	-----	-----
Surgery	Yes	0.33 (0.16 – 0.71)	0.004	0.47 (0.21 – 1.08)	0.07
	None^*^	-----	-----	-----	-----
Intravenous accesses	Peripheral line	1.57 (0.85 – 2.91)	0.15	0.86 (0.32 – 2.28)	0.76
	Central line^*^	-----	-----	-----	-----
Total parental nutrition	Yes	0.63 (0.34 – 1.14)	0.12	0.87 (0.33 – 2.31)	0.79
	None^*^	-----	-----	-----	-----

Table 6 also examines the relationship between respiratory infection and potential risk factors. It demonstrates that invasive ventilation and cardiovascular co-morbidities significantly elevate the risk of respiratory infection, with odds ratios of 5.96 (2.08 - 17.11) and 1.97 (1.09 - 3.57), respectively. Furthermore, Table 7 shows that previous surgery significantly increases the risk of wound swab infection (OR= 10.71 (3.50- 32.77)).

**Table 6:** Results of the multivariate analysis of potential risk factors and respiratory tract infection * Reference category

Variables	Subgroups	Crude Odds Ratio (95% CI)	P-value	Adjusted Odds Ratio (95% CI)	P-value
Gestational age (weeks)	24-28	4.64 (2.25 – 9.59)	<0.001	0.50 (0.08 – 2.96)	0.44
	29-32	1.09(0.46 – 2.55)	0.85	0.87 (0.15 – 4.91)	0.87
	33-36	1.45(0.57 – 3.73)	0.44	1.03 (0.19 – 5.61)	0.97
	≥ 37^*^	-----	-----	-----	-----
Birth weight (grams)	<999	6.37 (2.92 – 13.90)	<0.001	2.24 (0.37 – 13.67)	0.38
	1000-1499	0.55 (0.17 – 1.75)	0.31	0.19 (0.03 – 1.41)	0.11
	1500-2499	1.96 (0.85 – 4.52)	0.12	1.24 (0.25 – 6.02)	0.79
	≥2500^*^	-----	-----	-----	-----
Chromosomal anomalies	Yes	0.36 (0.16 – 0.81)	0.01	0.45 (0.15 – 1.36)	0.16
	None^*^	-----	-----	-----	-----
Invasive ventilation	Yes	11.74 (4.90 – 28.13)	<0.001	5.96 (2.08 – 17.11)	<0.001
	None^*^	-----	-----	-----	-----
Non-invasive ventilation	Yes	1.66 (0.81 – 3.37)	0.17	0.82 (0.33 – 2.05)	0.68
	None^*^	-----	-----	-----	-----
Cardiovascular comorbidities	Yes	2.93 (1.79 – 4.80)	<0.001	1.97 (1.09 – 3.57)	0.03
	None^*^	-----	-----	-----	-----
Surgery	Yes	1.71 (1.02 – 2.88)	0.04	1.25 (0.67 – 2.31)	0.49
	None^*^	-----	-----	-----	-----
Intravenous accesses	Peripheral line	0.19 (0.09 – 0.40)	<0.001	0.36 (0.12 – 1.08)	0.07
	Central line^*^	-----	-----	-----	-----
Total parental nutrition	Yes	6.33 (3.01 – 13.33)	<0.001	1.83 (0.62 – 5.35)	0.27
	None^*^	-----	-----	-----	-----

**Table 7:** Results of the multivariate analysis of potential risk factors and wound swab infection * Reference category

Variables	Subgroups	Crude Odds Ratio (95% CI)	P-value	Adjusted Odds Ratio (95% CI)	P-value
Gestational age (weeks)	24-28	0.21(0.06 – 0.71)	0.01	0.23 (0.01 – 4.79)	0.34
	29-32	0.61(0.20 – 1.86)	0.38	1.25 (0.15 – 10.72)	0.83
	33-36	1.33(0.44 – 4.01)	0.62	1.36 (0.25 – 7.32)	0.72
	≥ 37^*^	-----	-----	-----	-----
Birth weight (grams)	<999	0.31(0.09 – 1.01)	0.05	5.97 (0.28 – 129.76)	0.25
	1000-1499	0.48(0.12 – 1.97)	0.31	1.54 (0.11 – 21.08)	0.73
	1500-2499	0.99(0.35 – 2.87)	0.98	1.05 (0.20 – 5.54)	0.96
	≥2500^*^	-----	-----	-----	-----
Chromosomal anomalies	Yes	3.99 (1.63 – 9.76)	0.002	2.90 (0.85 – 9.94)	0.09
	None^*^	-----	-----	-----	-----
Invasive ventilation	Yes	0.39 (0.17 – 0.88)	0.02	0.26 (0.07 – 0.99)	0.049
	None^*^	-----	-----	-----	-----
Non-invasive ventilation	Yes	0.41 (0.16– 1.06)	0.07	0.81 (0.25 – 2.63)	0.73
	None^*^	-----	-----	-----	-----
Cardiovascular comorbidities	Yes	0.74 (0.32– 1.70)	0.47	-----	-----
	None^*^	-----	-----	-----	-----
Surgery	Yes	5.31 (2.24– 12.57)	<0.001	10.71 (3.50– 32.77)	<0.001
	None^*^	-----	-----	-----	-----
Intravenous accesses	Peripheral line	2.30 (0.98– 5.38)	0.06	0.72 (0.19– 2.72)	0.62
	Central line^*^	-----	-----	-----	-----
Total parental nutrition	Yes	0.20 (0.09– 0.47)	<0.001	0.22 (0.06– 0.84)	0.03
	None^*^	-----	-----	-----	-----

**Figure 1:**
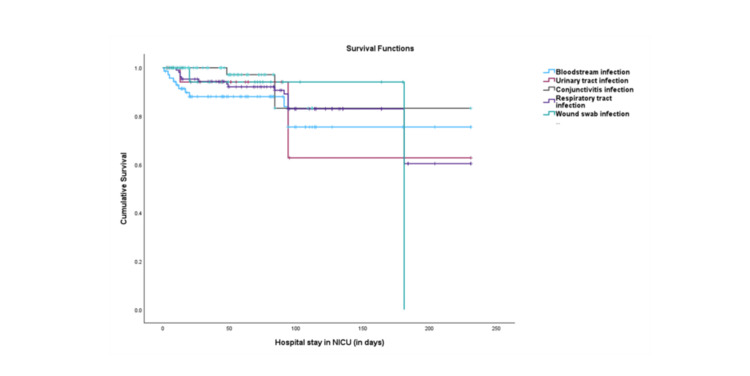
Kaplan-Meier curves of survival rates among NI NI: nosocomial infections

The Kaplan-Meier survival curves, depicted in Figure 1, illustrate the survival distribution among the different types of nosocomial infections. Our findings suggest that bloodstream infections (15.5%) and respiratory tract infections (15.9%) are associated with higher mortality rates compared to other infections; however, the differences are not statistically significant (P=0.14). Furthermore, respiratory tract infections had the highest median length of hospital stay at 94 (10-231) days, and the difference in length of stay between the different types of infections was statistically significant (P<0.001) (Table 8). However, there is no statistically significant difference in survival rates among the various types of infections (Figure 1).

**Table 8:** Results of the total length of hospital stay * chi-square test and Kruskal-Wallis test

NI								Test statistic value	P-value
Variables	Subgroups	Bloodstream infection (N=71)	Urinary tract Infection (N=22)	CNS (N=2)	Conjunctivitis (N=65)	Respiratory tract infection (N=107)	Surgical site infection (wound swabs) (N=25)		
Survival	Alive	60 (84.5)	20 (90.9)	2 (100)	63 (96.9)	90 (84.1)	23 (92.0)	8.26	0.14^*^
	Dead	11 (15.5)	9 (9.1)	0 (0)	2 (3.1)	17 (15.9)	2 (8.0)		
Hospital stays (days), Median (min-max)		53 (1-231)	48 (3-231)	42 (20-64)	50 (3-231)	94 (10-231)	44 (5-181)	49.70	<0.001^&^

## Discussion

This retrospective cohort study, conducted at the NICU of SQUH over four years, provides a comprehensive analysis of NI in neonates. To the authors' knowledge, this is the first study to provide data on the prevalence, incidence, and risk factors of NI in neonates in Oman. The study found that the average overall prevalence of NI among neonates admitted to the NICU was 17.95%, with an average incidence of 13.75 NI per 1,000 patient-days and 17.78 NI per 100 admissions. The incidence of NI in neonates is notably high, but it still falls within the range of reported incidence rates worldwide [6,13-15]. The wide variation observed in the reported incidence of NI among neonates can be attributed to the differences in study design, population characteristics, and diagnostic criteria employed across the various studies.

The most prevalent NI in our study was respiratory tract infections (N=107), followed by bloodstream infections (N=71), a finding consistent with studies conducted in other NICUs [6,16,17]. The high prevalence of respiratory tract infections may be attributed to the characteristics of the study population, as the majority of the included neonates were both extremely preterm and had extremely low birth weights. Additionally, the widespread use of invasive procedures among neonates in the NICU may have contributed to the increased risk of respiratory infections.

The study's findings on the distribution of causative organisms align with those of other studies. Bloodstream infections were more frequently associated with Gram-positive bacteria, particularly *Staphylococcus epidermidis* (66.67%). The predominance of Gram-positive bacteria in bloodstream infections has been widely reported in the literature [18,19], underscoring the need for stringent infection control measures and judicious use of antimicrobial agents in this vulnerable patient population. In contrast, urinary tract infections, conjunctivitis, respiratory tract infections, and surgical site infections were more commonly caused by Gram-negative organisms. Research has emphasized the prominent role of Gram-negative bacteria as causative agents of NI, as evidenced by the findings of Zaidi et al. [20]. Maternal intrapartum antibiotic prophylaxis might explain the positive correlation of maternal antibiotics as a risk factor with Gram-negative infections [11].

The study identified several significant risk factors for NI in neonates. Notably, mechanical ventilation was found to be a significant risk factor for respiratory tract infections. This is consistent with existing literature that highlights the association between mechanical ventilation and an increased risk of respiratory infections in neonates [21-23]. Furthermore, the study found that previous surgery was a significant risk factor for surgical site infections, a finding that is consistent with the existing literature [24]. Other studies also highlighted low birth weight and prematurity as risk factors [18,25,26]. The absence of statistically significant variables influencing the occurrence of bloodstream infections and urinary tract infections in our study highlights the complex interplay of factors contributing to these infections in neonates, including quality of patient care, heterogeneity in clinical practices, differences in pathogen virulence, and sampling limitations. Our study found that cardiovascular co-morbidities were a significant risk factor for respiratory and urinary tract infections in neonates. This highlights a potential vulnerability in this population. Further research is needed to understand the underlying mechanisms and explore ways to prevent infections in neonates with cardiovascular conditions.

The Kaplan-Meier survival analysis revealed that respiratory tract infections and bloodstream infections were associated with lower survival rates, although the difference was not statistically significant. This finding could be because the sample size was not large enough to detect significant differences. Respiratory tract infections were also associated with significantly longer hospital stays, aligning with findings from several other studies [27,28].

This study provides the first assessment of the burden of NI infections, including their epidemiological characteristics and associated factors, in Oman. The study had a few limitations that are worth mentioning. First, the study was conducted at a single center, which may limit the generalizability of the findings. Second, inherent to the retrospective study design, the reliance on pre-existing medical records introduced the potential for information bias due to missing or incomplete data, and the accuracy of infection ascertainment could have been influenced by variations in documentation practices across the four-year study period. Third, the absence of molecular typing of pathogens limited our ability to identify potential outbreak clusters or resistance patterns. This could have provided a more in-depth analysis of the transmission dynamics within the NICU. Finally, the lack of a control group prevents the determination of causality and makes it challenging to compare the incidence of NI in our study population to that of a similar population without the same exposures and interventions.

Despite these limitations, the study provides valuable insights into the epidemiology of NI in neonates in Oman and highlights the need for effective infection control measures in NICUs.

## Conclusions

These findings underscore the urgent need for the implementation of evidence-based infection prevention protocols and enhanced surveillance systems in Omani NICUs. The predominance of Gram-positive organisms in bloodstream infections and Gram-negative organisms in respiratory tract infections further informs empiric antibiotic strategies. Further prospective studies with larger sample sizes are warranted to validate our findings and explore additional risk factors for NI in neonates.
